# The effect of starvation and re-feeding on mitochondrial potential in the midgut of *Neocaridina davidi* (Crustacea, Malacostraca)

**DOI:** 10.1371/journal.pone.0173563

**Published:** 2017-03-10

**Authors:** Agnieszka Włodarczyk, Lidia Sonakowska, Karolina Kamińska, Angelika Marchewka, Grażyna Wilczek, Piotr Wilczek, Sebastian Student, Magdalena Rost-Roszkowska

**Affiliations:** 1 University of Silesia, Department of Animal Histology and Embryology, Katowice, Poland; 2 University of Silesia, Department of Animal Physiology and Ecotoxicology, Katowice, Poland; 3 Heart Prosthesis Institute, Bioengineering Laboratory, Zabrze, Poland; 4 Silesian University of Technology, Institute of Automatic Control, Faculty of Automatic Control, Electronics and Computer Science, Gliwice, Poland; Instituto Nacional de Salud Pública, MEXICO

## Abstract

The midgut in the freshwater shrimp *Neocaridina davidi* (previously named *N*. *heteropoda*) (Crustacea, Malacostraca) is composed of a tube-shaped intestine and a large hepatopancreas that is formed by numerous blind-ended tubules. The precise structure and ultrastructure of these regions were presented in our previous papers, while here we focused on the ultrastructural changes that occurred in the midgut epithelial cells (D-cells in the intestine, B- and F- cells in the hepatopancreas) after long-term starvation and re-feeding. We used transmission electron microscopy, light and confocal microscopes and flow cytometry to describe all of the changes that occurred due to the stressor with special emphasis on mitochondrial alterations. A quantitative assessment of cells with depolarized mitochondria helped us to establish whether there is a relationship between starvation, re-feeding and the inactivation/activation of mitochondria. The results of our studies showed that in the freshwater shrimp *N*. *davidi* that were analyzed, long-term starvation activates the degeneration of epithelial cells at the ultrastructural level and causes an increase of cells with depolarized (non-active) mitochondria. The process of re-feeding leads to the gradual regeneration of the cytoplasm of the midgut epithelial cells; however, these changes were observed at the ultrastructural level. Additionally, re-feeding causes the regeneration of mitochondrial ultrastructure. Therefore, we can state that the increase in the number of cells with polarized mitochondria occurs slowly and does not depend on ultrastructural alterations.

## Introduction

During long-term periods of starvation, animals increase their ability to survive by changing the activity of the digestive system. Response to starvation (as a stress factor) is manifested through changes at the physiological, biochemical and molecular levels and can mainly be observed in all of the organs that are responsible for the storage of the reserve material, e.g. the midgut epithelium [1–3]. Re-feeding may ultimately result in changes such as the elongation of intestinal villi, the elongation of microvilli or the shortening of the intestine length. Therefore, the organism can regulate its homeostasis [4]. Numerous changes can be observed at the ultrastructural level, e.g. in mitochondria, which are organelles that are responsible for the production of ATP, or the synthesis of steroids or reactive oxygen species (ROS). Mitochondria are also responsible for the activation of the cell death processes [5,6]. Numerous studies revealed that a larger number of these organelles is connected with a higher demand for energy in the cell [7,8]. Measurements of the transmembrane mitochondrial potential (ΔΨm) are treated as markers of any changes that could lead to any ultrastructural level and eventually, to cell death [9–11]. JC-1 (5,5ʹ,6,6ʹ-tetrachloro-1,1ʹ,3,3ʹ-tetraethyl-benzimidazolyl-carbocyanine iodide), which is a membrane-permeant cationic dye, is the most suitable chemical reagent that can be used in these studies because it can detect both active and inactive mitochondria in the same cell. Therefore, it is usually used in studies of mitochondrial activity in cells and tissues [10,11].

The freshwater shrimp *Neocaridina davidi* (previously called *Neocaridina heteropoda)* comes from Taiwan. It is popular because of the ease of culturing and obtaining them (from local breeders). The ultrastructure of the digestive system, with an emphasis on its midgut, was described in our previous papers [11,12]. The midgut of this species is composed of a tube-shaped intestine and a large gland, which is called the hepatopancreas. Both organs are lined with a monolayered epithelium that rests on a non-cellular basal lamina. The epithelium of the intestine is formed by two types of cells—the digestive (D-cells) and the regenerative cells (E-cells). The latter are scattered in the anterior region of the intestine. The hepatopancreas is a lobular organ that is formed by two large diverticules, which are secondarily divided into blind-end tubules. Each tubule is divided into three regions—a distal zone with regenerative cells (E-cells) in its epithelium, a median zone with differentiating cells and a proximal region with fibrillar (F-cells, fibrillar cells) and storage cells (B-cells, storage cells) [12].

**Fig 1:**
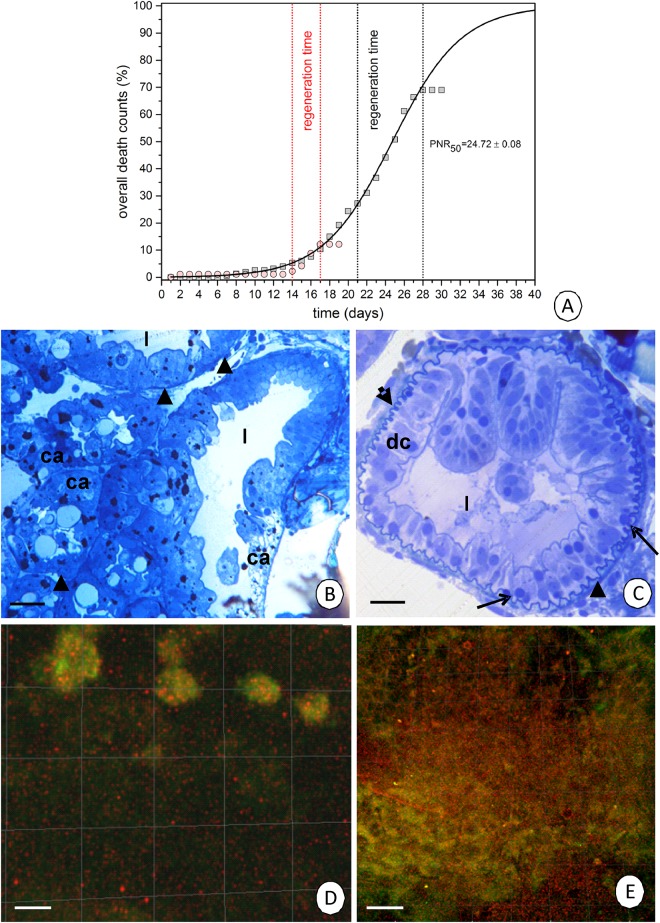
**(A)** The diagrammatic representation of shrimps’ survival according to starvation and re-feeding. **(B-E)** The midgut of non-starved specimens of *N*. *davidi*. **(B)** A longitudinal section through the hepatopancreas with numerous caeca (ca). Light microscopy. Bar = 15 μm. Midgut lumen (l), basal lamina (arrowheads). **(C)** A transverse section through the anterior fragment of the intestine with regenerative cells (arrows) distributed among the basal regions of the digestive cells (dc). Light microscopy. Bar = 23 μm. **(D-E)** Mitochondrial potential in the *N*. *davidi* midgut epithelium. Active mitochondria with a high membrane potential (red signals), inactive mitochondria with a low membrane potential (green signals). JC-1 cationic dye. Confocal microscope. **(D)** Hepatopancreas in non-starved animals. Bar = 10 μm. **(E)** Intestine in non-starved animals. Bar = 8 μm.

The aim of these studies was to perform an experiment with adult specimens of *N*. *davidi* that were starved for one, two or three weeks. The periods of starvation were selected according to a survival graph (Fig 1A). After the periods of starvation, the animals were re-fed. The experiment permits the changes in the D-cells (intestine), F- and B-cells (hepatopancreas) of the midgut epithelium that occurred after starvation and re-feeding to be described at the ultrastructural level with special emphasis on the ultrastructure of mitochondria, which are treated as structures that are sensitive to stressors [13]. Therefore, we could determine whether the changes in the cells and organelles are reversible or not.

### Results

#### Point of No-Return (PNR_50_)—Regeneration

The point of no-return (PNR_50_) was equal to 24.72 ±0.08. For the shrimp that were starved continuously for 14 days, the regeneration period was equal to three days, while for the shrimp that were starved for 21 days (close to the PNR_50_), the regeneration period was extended to seven days but was still possible (Fig 1A). No differences were observed between males and females, and therefore the description concerns both sexes. Additionally, the description is connected with the B- and F-cells in the hepatopancreas (Fig 1B) and D-cells in the intestine (Fig 1C) because no changes were observed in the E-cells of either organ.

#### Mitochondrial potential in the intestine and hepatopancreas

Application of the membrane-permeant JC-1 cationic dye indicated that the active mitochondria that had a high membrane potential showed a red fluorescence, while a green fluorescence of the monomeric form of the dye indicated the localization of inactive mitochondria that had a low membrane potential. The average percentage of cells with depolarized mitochondria was low in both organs of the control specimens [11]– 3.93 ±2.7% in the hepatopancreas (Fig 1D), while it was 5.13 ± 3.5% in the intestine (Fig 1E). There were no statistically significant differences in the level of this parameter between the organs that were analyzed [11].

**Fig 2:**
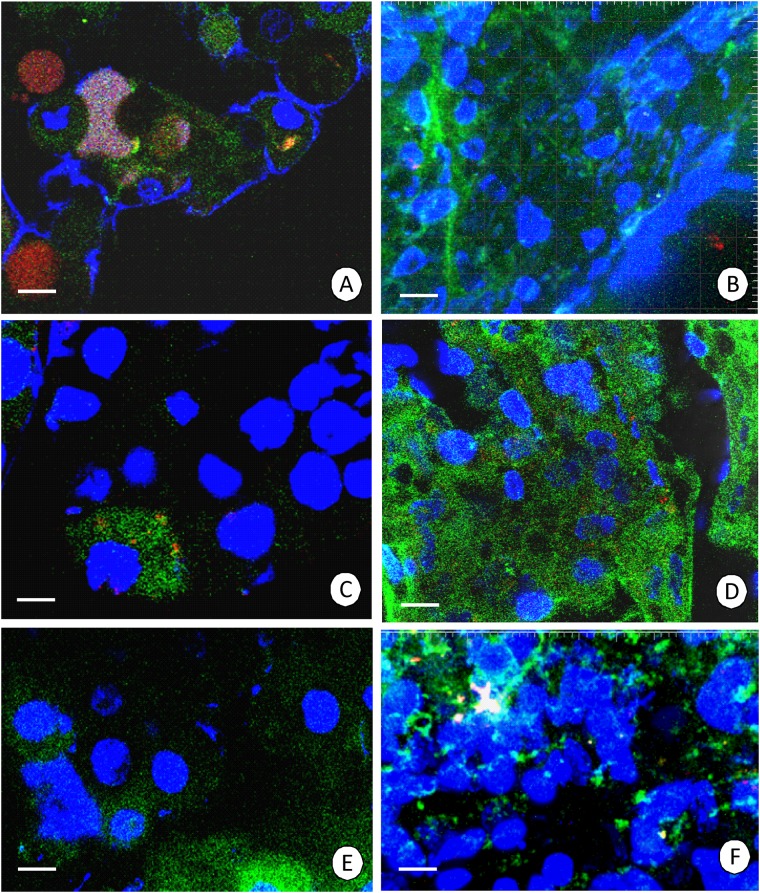
Mitochondrial potential in the *N*. *davidi* midgut epithelium. Active mitochondria with a high membrane potential (red signals), inactive mitochondria with a low membrane potential (green signals), nuclei (blue signals). JC-1 cationic dye, Hoechst 33342 staining. Confocal microscope. **(A)** Hepatopancreas in animals starved for 7 days. Bar = 10 μm. **(B)** Intestine in animals starved for 7 days. Bar = 5 μm. **(C)** Hepatopancreas in animals starved for 14 days. Bar = 5 μm. **(D)** Intestine in animals starved for 14 days. Bar = 10 μm. **(E)** Hepatopancreas in animals starved for 21 days. Bar = 5 μm. **(F)** Intestine in animals starved for 21 days. Bar = 5 μm.

In the shrimp that were starved for seven days, the average percentage of cells with depolarized mitochondria was 24.3 ± 4.6% in the hepatopancreas (Fig 2A), (S1 Video), while it was 22.4 ± 4.0% in the intestine (Fig 2B), (S2 Video). After 14 days (two weeks) of starvation, the average percentage of cells with depolarized mitochondria was 41.5 ± 13.3% in the hepatopancreas (Fig 2C), (S3 Video), while it was 31.8 ± 13.8% in the intestine (Fig 2D), (S4 Video). After 21 days (three weeks) of starvation, the average percentage of cells with depolarized mitochondria was 40.3 ± 12.5% in the hepatopancreas (Fig 2E), (S5 Video), while it was 32.8 ± 7.5% in the intestine (Fig 2F), (S6 Video).

The animals that were starved for seven days were not re-fed and analyzed because no ultrastructural alterations were visible in the cytoplasm and organelles (see below in the text). After the re-feeding the shrimp that were staved for 14 and 21 days, the changes in the number of depolarized mitochondria were similar, and therefore the results that are presented below concern the animals that were starved for 14 days (two weeks) and then re-fed. Additionally, the analysis of animals that were starved for 21 days (three weeks) was difficult because the time of the starvation was equal to the PNR_50_ point.

**Fig 3:**
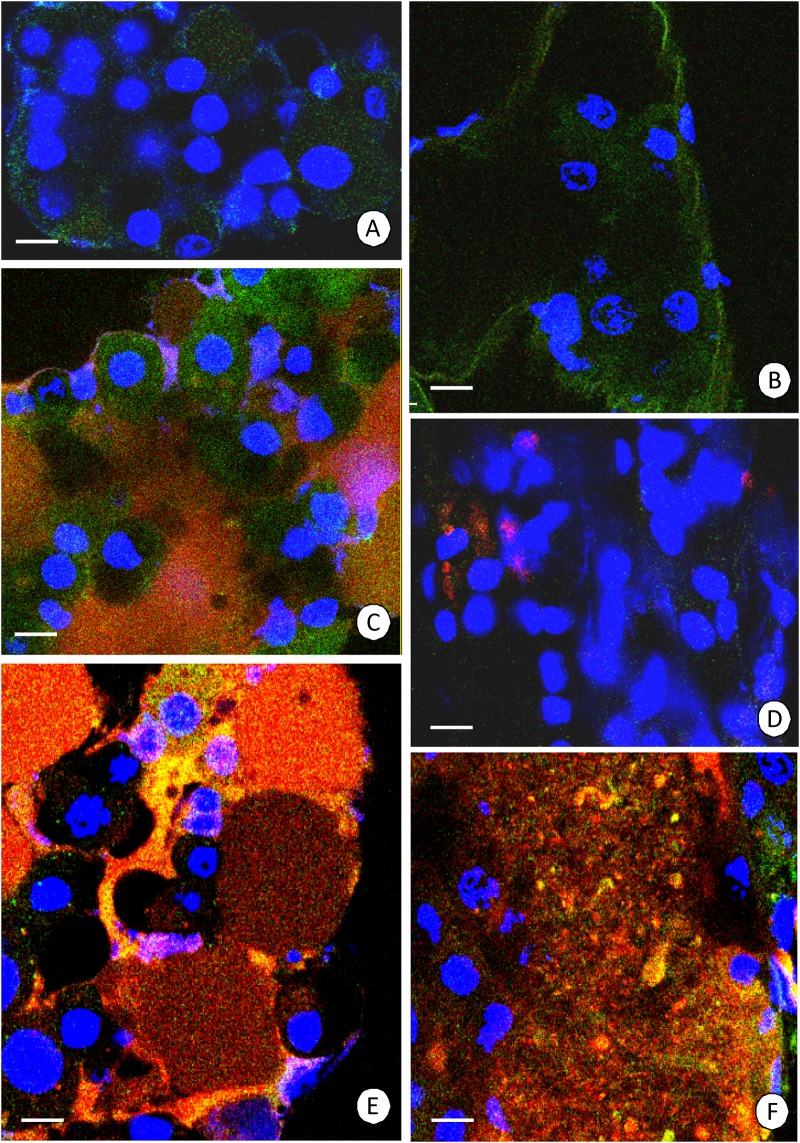
Mitochondrial potential in the *N*. *davidi* midgut epithelium. Active mitochondria with a high membrane potential (red signals), inactive mitochondria with a low membrane potential (green signals), nuclei (blue signals). JC-1 cationic dye, Hoechst 33342 staining. Confocal microscope. **(A)** Hepatopancreas in animals re-fed for 4 days after 14 days of starvation. Bar = 5 μm. **(B)** Intestine in animals re-fed for 4 days after 14 days of starvation. Bar = 5 μm. **(C)** Hepatopancreas in animals re-fed for 7 days after 14 days of starvation. Bar = 5 μm. **(D)** Intestine in animals re-fed for 7 days after 14 days of starvation. Bar = 5 μm. **(E)** Hepatopancreas in animals re-fed for 14 days after 14 days of starvation. Bar = 5 μm. **(F)** Intestine in animals re-fed for 14 days after 14 days of starvation. Bar = 10 μm.

**Fig 4:**
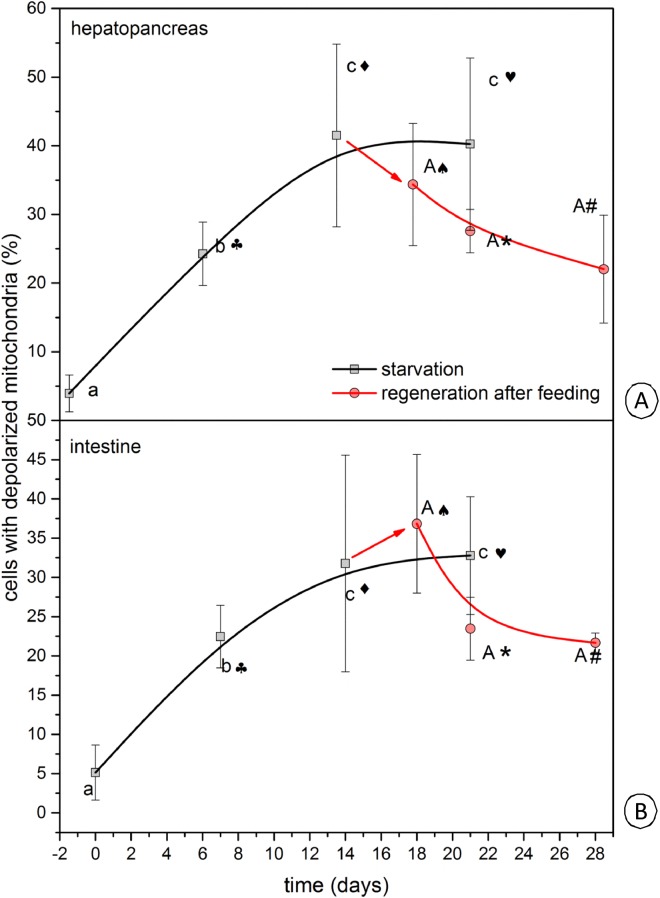
A diagrammatic representation of the average percentage of cells with depolarized mitochondria in the hepatopancreas (A) and intestine (B) in *N*. *davidi* exposed to starvation and after refeeding. *a*, *b*, *c*– starvation, *A*–re-feeding—determination whether the outcome of each step varies in a statistically significant way from the previous step. The same letter symbols represent no significant differences. Different graph symbols (♠♣♦♥*#) mean that the results differ significantly from the result under control.

After four days of re-feeding after 14 days of starvation, the average percentage of cells with depolarized mitochondria was 34.4 ± 8.9% in the hepatopancreas (Fig 3A), (S7 Video), while it was 36.8 ± 8.8% in the intestine (Fig 3B), (S8 Video). After seven days (one week) of re-feeding the shrimp that were starved for 14 days, the average percentage of cells with depolarized mitochondria was 27.6 ± 3.2% in the hepatopancreas (Fig 3C), (S9 Video), while it was 23.5 ± 4.0% in the intestine (Fig 3D), (S10 Video). In the shrimp that were starved for 14 days that were re-fed for 14 days (two weeks), the average percentage of cells with depolarized mitochondria was 22.0 ± 7.8% in the hepatopancreas (Fig 3E), (S11 Video), while it was 21.7 ± 1.2% in the intestine (Fig 3F), (S12 Video). After 21 days of re-feeding, no changes appeared with respect to 14 days of re-feeding (not shown). The number of active mitochondria in the intestine and hepatopancreas increased in a similar manner (Fig 4A and 4B, Table 1).

**Table 1:** Percentage [%] of intestinal and hepatopancreatic epithelial cells with active mitochondria in the *N*. *davidi* that were re-fed after 14 days of starvation.

Number of days of re-feeding after 14 days of starvation	Percentage [%] of cells with active mitochondria	
	Intestine	Hepatopancreas
**4 days of re-feeding**	63.2 ± 8.8%	65.6 ± 8.9%
**7 days of re-feeding**	76.5 ± 4%	72.4 ± 3.2%
**14 days of refeeding**	78 ± 7.8%	78.3 ± 1.2%

#### Fine structure of the midgut epithelium—Ultrastructure of mitochondria

**Fig 5:**
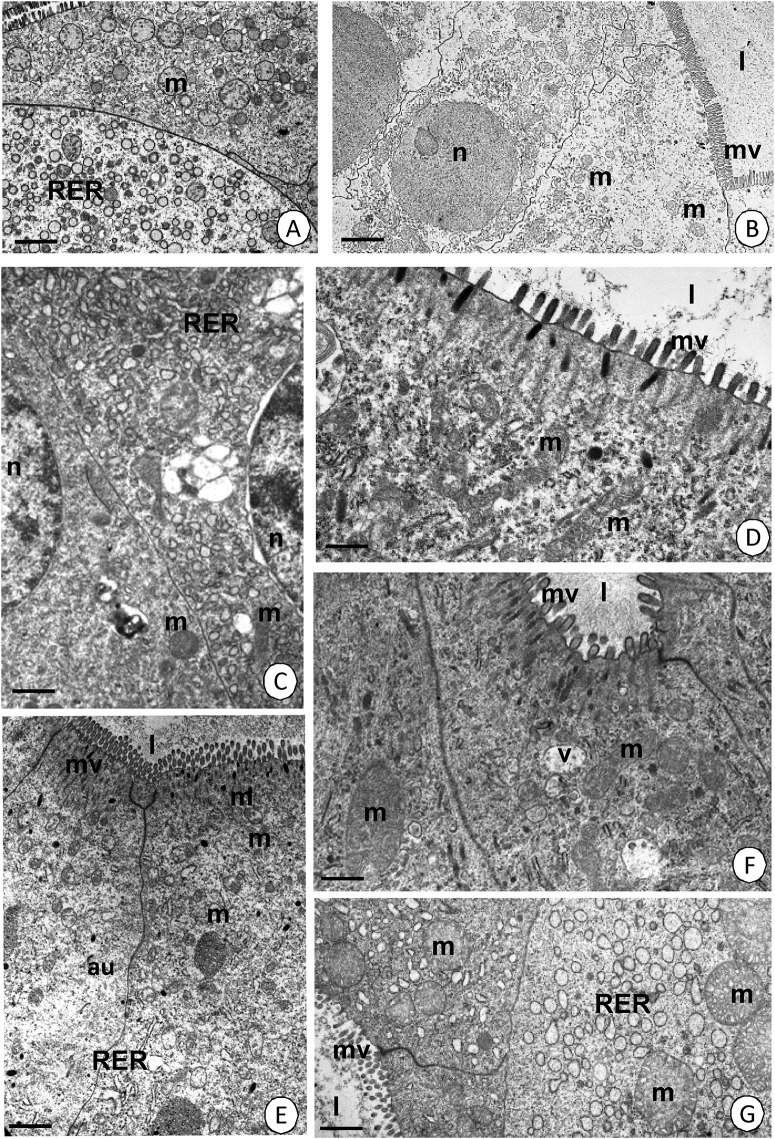
Ultrastructure of the midgut epithelium in *N*. *davidi*. TEM. Mitochondria (m), midgut lumen (l), microvilli (mv), nucleus (n), cisterns of RER (RER), vacuoles (v), autophagosomes (au). **(A)** Hepatopancreas in non-starved animals. Bar = 1.5 μm. **(B)** Intestine in non-starved animals. Bar = 1 μm. **(C)** Hepatopancreas in animals starved for 7 days. Bar = 1 μm. **(D)** Intestine in animals starved for 7 days. Bar = 1 μm. **(E)** Hepatopancreas in animals starved for 14 days. Bar = 0.15 μm. **(F)** Intestine in animals starved for 14 days. Bar = 1 μm. **(G)** Hepatopancreas in animals starved for 14 days. Bar = 1.5 μm.

**Fig 6:**
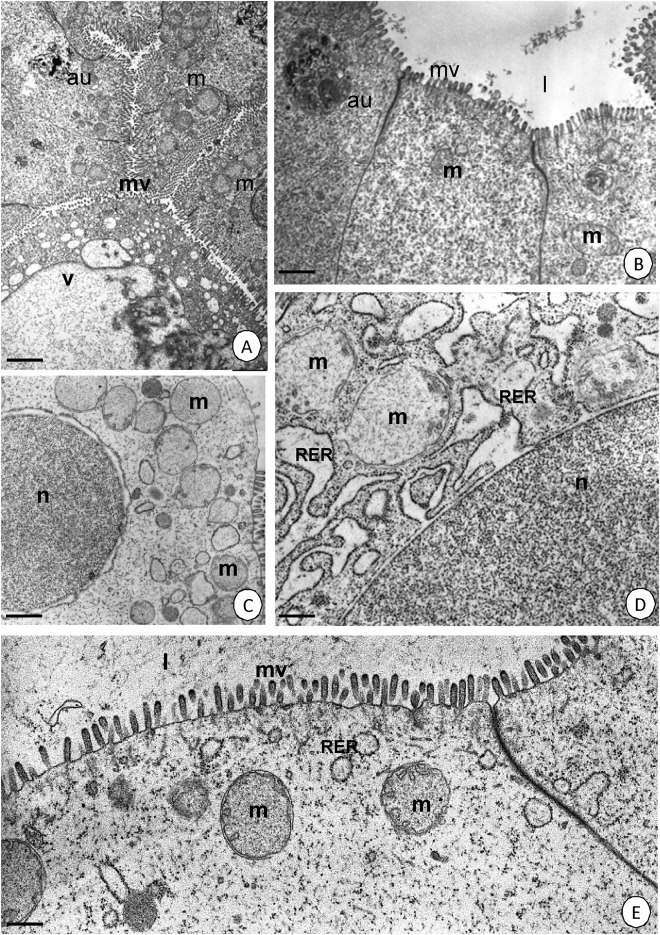
Ultrastructure of the midgut epithelium in *N*. *davidi*. TEM. Mitochondria (m), midgut lumen (l), microvilli (mv), nucleus (n), cisterns of RER (RER), vacuoles (v), autophagosomes (au). **(A)** Hepatopancreas in animals starved for 21 days. Bar = 2.6 μm. **(B)** Intestine in animals starved for 21 days. Bar = 1 μm. **(C)** Hepatopancreas in animals re-fed for 4 days after 14 days of starvation. Bar = 1.5 μm. **(D)** Intestine in animals re-fed for 4 days after 14 days of starvation. Bar = 0.5 μm. **(E)** Intestine in animals re-fed for 4 days after 14 days of starvation. Bar = 0.5 μm.

After seven days of starvation, the ultrastructure of the D-cells in the intestine and the F- and B-cells in the hepatopancreas of *N*. *davidi* did not change compared to the control group, which was described in our previous papers [11,12] (Fig 5A and 5B). The typical structure of mitochondria had a medium electron-dense matrix and many long cristae (Fig 5C and 5D). After 14 days (two weeks) of starvation, neither the cytoplasm of the D-, B- and F-cells or the ultrastructure of mitochondria changed (Fig 5E). However, some bloated mitochondria with a decreased number of short cristae appeared. Additionally, in some places, the cytoplasm began to be poor in organelles such as the rough and smooth endoplasmic reticulum (Fig 5F and 5G). Twenty-one days of starvation (three weeks) led to the appearance of an electron-lucent cytoplasm in the D-, F- and B-cells of the midgut epithelium. The number of organelles (e.g. rough endoplasmic reticulum) decreased significantly, and therefore the cytoplasm was poor in these structures (Fig 6A). Additionally, the majority of the bloated mitochondria were degenerated; they had an electron-lucent matrix and only a few short cristae (Fig 6B). We can conclude that the process of starvation caused the gradual degeneration of mitochondria as well as the decrease of organelles the in D-, F- and B-cells of the midgut.

Because the specimens that were starved for seven days (one week) showed no ultrastructural changes in the D-, B- and F-cells of the midgut epithelium, no regeneration experiment was performed on them. Additionally, no ultrastructural alterations in the cytoplasm of the D-, F- and B-cells of the midgut epithelium appeared after four days of re-feeding the shrimp that were starved for 14 or 21 days, and therefore the cytoplasm was still as poor in organelles as it was after the period of starvation. Additionally, mitochondria were still expanded (Fig 6C, 6D and 6E). However, a larger number of the rough endoplasmic reticulum appeared (Fig 6C).

**Fig 7:**
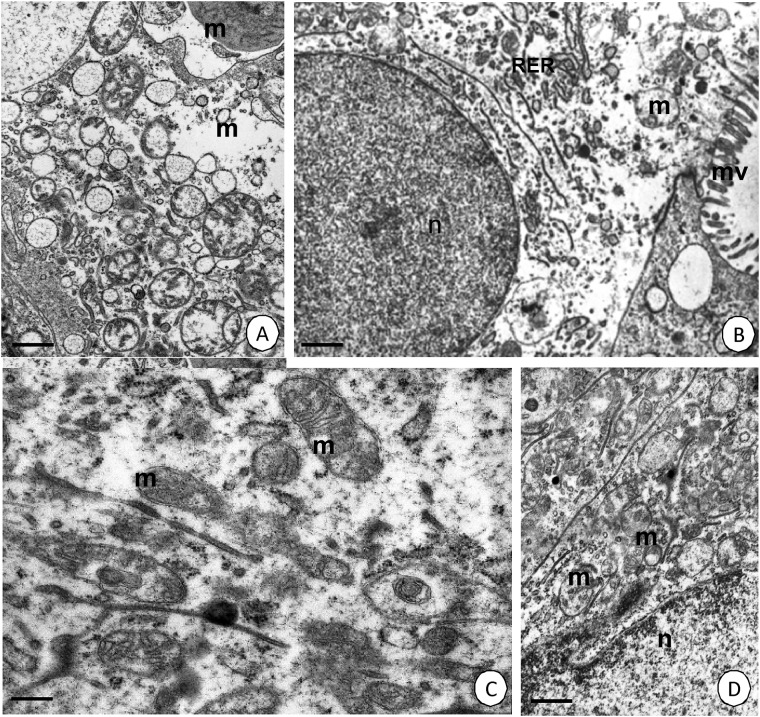
Ultrastructure of the midgut epithelium in *N*. *davidi*. TEM. Mitochondria (m), midgut lumen (l), microvilli (mv). **(A)** Hepatopancreas in animals re-fed for 7 days after 14 days of starvation. Bar = 1 μm. **(B)** Intestine in animals re-fed for 7 days after 14 days of starvation. Bar = 1 μm. **(C)** Hepatopancreas in animals re-fed for 14 days after 14 days of starvation. Bar = 0.5 μm. **(D)** Intestine in animals re-fed for 14 days after 14 days of starvation. Bar = 1 μm.

Re-feeding the shrimp that were starved for 14 days (two weeks) for seven days (one week) caused the cytoplasm of the D-, B- and F-cells to still have an electron-lucent cytoplasm that was poor in organelles. However, mitochondrial cristae began to elongate in the bloated mitochondria. The cisterns of the rough endoplasmic reticulum also started to accumulate (Fig 7A and 7B). In specimens that were re-fed for 14 days (two weeks) after 14 days (two weeks) of starvation, the cytoplasm of the D-, B- and F-cells began to be electron-dense and rich in organelles that were characteristic of these cells in the control group. mitochondria had long cristae and a matrix that was of a medium electron density (Fig 7C and 7D). The cytoplasm of the D-, F- and B-cells of the midgut epithelium resembled the ultrastructure of these cells in the control group. Therefore, we can conclude that two weeks of re-feeding were sufficient to regenerate the ultrastructure and mitochondria in the D-, B- and F-cells in the midgut epithelium of *N*. *davidi*.

The ultrastructure of the D-, B- and F-cells in the midgut epithelium of the shrimp that were re-fed for 7, 14 and 21 days after 21 (three weeks) of starvation had the same changes as those described for the animals that were starved for 14 days (not shown). The process of re-feeding after periods of starvation caused the gradual self-renewal of the D-cells in the intestine and the B- and F-cells in the hepatopancreas.

### Discussion

When a lack of food occurs in the environment where an animal lives, numerous changes occur in the digestive system, which enable the animal to survive [14,15]. To date, many studies have been conducted on crustaceans that have been starved; however, they mainly concern the physiological alterations in the organisms and they were conducted on species that had adapted to short-term periods of starvation, e.g. the sea shrimp *Litopenaeus vannamei*. This species does not feed itself during the five days that it molts [16]. To show the survival of adult individuals of *N*. *davidi*, the point of no-return (PNR_50_), which is the point at which the mortality rate is so high that a subsequent regeneration of the body is no longer possible, was used. It was detected for 200 starved adult individuals and was equal to 24.72 days. This value is higher than the one that was determined for first developmental stages of this species that were described by Pantaleo et al. [17], who found that PNR_50_ was equal to 16 days for the first larval stage (just after hatching), while it was equal to nine days after two subsequent moltings. Young specimens utilized the reserve material that was accumulated in the yolk, because they are not able to feed themselves. When the reserve material was exploited, a rapid growth of the organism occurred and a great deal of energy needed to be supplied. Therefore, the mortality of starved shrimps occurred [17]. In the case of adult specimens of *N*. *davidi*, the demand for food is significantly lower than for larvae because growth and moltings are severely limited. Additionally, adult specimens can accumulate reserve material in the midgut epithelial cells (the B-cells of the hepatopancreas) [12] or even in the fat body [18]. Therefore, the length of survival is extended compared to younger specimens. Our studies revealed that the ultrastructural changes in the midgut epithelial cells of *N*. *davidi* are significant after three weeks of starvation. The number of degenerated organelles becomes so high that the animal dies, so a distinct relationship appears between the period of life after starvation and changes in the described epithelium at the ultrastructural level.

Mitochondria are multifunctional organelles that are responsible for the synthesis of ATP and reactive oxygen species (ROS) and participate in the cell death processes [19]. Numerous ultrastructural alterations of mitochondria have been described as being caused by starvation [20,21] or even as being induced by other stressors [22–25]. During their degeneration, mitochondria might be swollen or contracted, their cristae may shorten and eventually disappear. mitochondrial matrix becomes electron dense or electron lucent [24, 26,27]. Degenerated mitochondria can be neutralized in a type of selective autophagy that is called mitophagy [24, 27–29]. Changes in the ultrastructure, including changes in the structure of the cristae, together with changes of mitochondrial transmembrane potential (ΔΨm) may be connected with the activation of the cell death, e.g. apoptosis [11]. Secretion of cytochrome c and the other cofactors from the inner mitochondrial membrane is connected with the reconstruction of mitochondrial cristae [30]. In addition, a reduction in their number is also a way of providing energy [21]. Our previous studies showed that in the freshwater shrimp *N*. *davidi* cells with depolarized mitochondria were detected in about 5% of the D-cells of the intestine and in about 3.9% of the F- and B-cells in the hepatopancreas. Mitochondria that were enclosed inside the autophagosomes were detected. This type of selective autophagy is called mitophagy [31]. Eventually, mitophagy protects the entire cell against the cellular degeneration that is caused by mitochondrial dysfunction [27,28]. In the control specimens of *N*. *davidi*, degenerating mitochondria were neutralized in the autophagosomes, and therefore the number of cells with depolarized mitochondria was quite low and eventually the process of apoptosis or necrosis was inhibited [11].

During our experiment, ultrastructural changes were observed in the D-cells in the intestine and the F- and B-cells in the hepatopancreas. The main alterations were connected with mitochondria. A prolonged duration of starvation caused an intensification in the degeneration of the cytoplasm. Re-feeding the animals that had been starved earlier caused the gradual regeneration of described cells—after four days no changes occurred, while after seven days of re-feeding, mitochondria started to change their ultrastructure. Therefore, we can state that this was the first regenerative process in the midgut epithelial cells. The cytoplasm was regenerated after 14 days of re-feeding. This suggests that mitochondria are the most sensitive organelles in cells [10, 23, 26], and that therefore, they are self-renewed as the first organelles [22, 26]. Despite the fact that the cytoplasm of the D-, F- and B-cells of the midgut epithelium resembled the ultrastructure of these cells in the control group after 14 days of re-feeding, the number of active mitochondria never reached the level of their activity in the control group. Therefore, we can conclude that two weeks of re-feeding were sufficient to regenerate the ultrastructure of the cells and the ultrastructure of mitochondria in the D-, B- and F-cells in the midgut epithelium of *N*. *davidi*. Changes in mitochondrial structure must have been so solid that not all of mitochondria restored their activity. It is worth noting that the degeneration of mitochondria can activate cell death (e.g. apoptosis, necrosis) [19, 23]. In *N*. *davidi*, the regeneration of mitochondria may prevent a cell against its death or changed mitochondria can activate cell death. This should be resolved during our further studies on the activation of cell death that is connected with starvation and re-feeding.

During our studies, using JC-1 dye confirmed the TEM analysis. A prolonged period of starvation caused the percentage of depolarized (non-active) mitochondria to gradually increase. Re-feeding induced the growth of the number of cells with active (polarized) mitochondria in the cells of the intestine and hepatopancreas and the mitochondrial membrane potential grew gradually. The increased number of mitochondria with a lower membrane potential at the beginning of starvation suggests an increasing number of degenerating cells [15, 32]. Simultaneously, after 14 days of starvation, although the period of starvation may be prolonged, the number of cells with depolarized mitochondria does not change. This may be connected with the fact that at this stage of starvation, the level of ATP is too low to activate apoptosis [33]. A decrease in the number of cells with non-active mitochondria in the intestine and hepatopancreas of *N*. *davidi* after re-feeding may be connected with the activation of autophagy (mitophagy, as the selective autophagy). The activation of autophagy due to starvation has been described as the process that protects the cell against the degradation of the proteins and organelles (e.g. mitochondria) that lead to cell death [1–3, 34]. After two weeks of re-feeding, the number of cells with depolarized mitochondria was still slightly higher than in the non-starved specimens of *N*. *davidi* (control specimens). This suggests that this period was not sufficient for the proper and complete regeneration of degenerated mitochondria, or even the activation of mitophagy in order to neutralize such mitochondria. Ultimately, we can state that despite the fact that the ultrastructural alterations show a complete regeneration of the cytoplasm of the cells, the analysis of the mitochondria membrane potential distinctly showed that not all mitochondria were regenerated or neutralized.

### Conclusions

The results of these studies showed that in the freshwater shrimp *N*. *davidi* that were analyzed: (a) starvation activates the degeneration of epithelial cells at the ultrastructural level; (b) starvation causes an increase of cells with depolarized (non-active) mitochondria; (c) re-feeding leads to a gradual regeneration of epithelial cells but at the ultrastructural level; (d) re-feeding induces the regeneration of mitochondrial ultrastructure; and (e) an increase in the number of cells with active (polarized) mitochondria proceeds slowly and does not correlate with the ultrastructural alterations.

## Materials and methods

### Materials

The research was conducted on adult males and females of the freshwater shrimp *Neocaridina davidi* (previously called *N*. *heteropoda*) (Crustacea, Malacostraca, Decapoda). The specimens were obtained from local shrimp breeders and kept in a main laboratory breed, i.e. 40 L shrimp tank equipped with a heater with a thermostat and a mechanical filtration system. The water temperature was set to 21°C, pH to 7 and total water hardness was equal to 10^0^d. The *N*. *davidi* shrimp were fed with JBL Novo Prawn.

#### Experiment

The starvation experiment was performed by placing the shrimp in isolated plastic (250 mL) containers. For the experiment, adult shrimp with a cephalothorax length above 2.5 mm were chosen. Every day 10% of the water was replaced and the plastic containers were cleared of excrements and cuticle exoskeletons. Containers were kept in a shaded room to avoid the development of algae. The shrimp were starved for 7, 14 and 21 days. Specimens from each experimental group were collected for the studies. Additionally, some specimens from each experimental group were re-fed for 4, 7, 14 and 21 days. The number of specimens from each experimental group that were collected for the experiment and all of the techniques that were used is presented in Table 2.

**Table 2:** Number of adult specimens of *N*. *davidi* that were used in each part of the experiment.

**Number of days of starvation**		**Number of specimens analyzed Part 1: starvation**		
		**TEM**	**Flow cytometry**	**Confocal microscopy**
7 days	males	5	24	4
	females	5	24	4
14 days	males	5	24	4
	females	5	24	4
21 days	males	5	24	4
	females	5	24	4
**Number of days of re-feeding after 7 days of starvation**		**Number of specimens analyzed Part 2: re-feeding after 7 days of starvation**		
		**TEM**	**Flow cytometry**	**Confocal microscopy**
4 days	males	5	24	4
	females	5	24	4
7 days	males	5	24	4
	females	5	24	4
14 days	males	5	24	4
	females	5	24	4
21 days	males	5	24	4
	females	5	24	4
**Number of days of re-feeding after 14 days of starvation**		**Number of specimens analyzed Part 3: re-feeding after 14 days of starvation**		
		**TEM**	**Flow cytometry**	**Confocal microscopy**
4 days	males	5	24	4
	females	5	24	4
7 days	males	5	24	4
	females	5	24	4
14 days	males	5	24	4
	females	5	24	4
21 days	males	5	24	4
	females	5	24	4
**Number of days of re-feeding after 21 days of starvation**		**Number of specimens analyzed Part 4: re-feeding after 21 days of starvation**		
		**TEM**	**Flow cytometry**	**Confocal microscopy**
4 days	males	5	24	4
	females	5	24	4
7 days	males	5	24	4
	females	5	24	4
14 days	males	5	24	4
	females	5	24	4
21 days	males	5	24	4
	females	5	24	4

### Methods

#### Light and transmission electron microscopy

Adult specimens of *N*. *davidi* were decapitated and fixed with 2.5% glutaraldehyde in a 0.1 M sodium phosphate buffer (pH 7.4, 4°C, 2h), postfixed in 2% osmium tetroxide in a 0.1 M phosphate buffer (4°C, 1.5 h) and dehydrated in a graded series of concentrations of ethanol (50, 70, 90, 95 and 4x100% each for 15 min) and acetone (15 min). Afterwards, the material was embedded in epoxy resin (Epoxy Embedding Medium Kit; Sigma). Semi- (0.8 μm thick) and ultra-thin (70 nm) sections were cut on a Leica Ultracut UCT25 ultramicrotome. Semi-thin sections were stained with 1% methylene blue in 0.5% borax and observed using an Olympus BX60 light microscope. After staining with uranyl acetate and lead citrate, ultra-thin sections were examined using a Hitachi H500 transmission electron microscope.

#### Preparation of cell suspension

For the flow cytometry study, the dissected organs (hepatopancreas and intestine that were isolated from five specimens for each experimental stage, i.e. different starvation durations and regenerations) were crushed mechanically and suspended in 100 μL PBS (pH 7.4). Then, using 0.05% trypsin in an EDTA solution (0.2 g ∙ L^-1^ EDTA in Hank’s Balanced Salt Solution) with 0.01% collagenase II, enzymatic isolation was carried out for 10 min at 37°C. The cells were suspended in a DMEM low-glucose medium (1g ∙ L^-1^) and incubated at 37°C. The cell suspension was washed using centrifugation at 1500 rpm for five minutes and the precipitate was suspended in 100 μL of a PBS buffer.

#### Quantitative assessment of cells with depolarized mitochondria

JC-1 (5,5ʹ,6,6ʹ-tetrachloro-1,1ʹ,3,3ʹ-tetraethyl-benzimidazolyl-carbocyanine iodide) is a membrane-permeant cationic dye that is widely used in order to monitor mitochondria in cell death studies. Changes in mitochondrial transmembrane potential (ΔΨm) were monitored using JC-1 cationic dye whose accumulation in mitochondria is dependent on the magnitude of mitochondrial potential. JC-1 differentiates cells with a high mitochondrial potential (orange fluorescence; polarized mitochondria) and a low mitochondrial potential (green fluorescence; depolarized mitochondria) [35]. The intestine and hepatopancreas were isolated from animals’ bodies (Table 2). The cell suspension obtained from these organs without fixation was incubated in the dark with 5 μL of the 1.5 mM JC-1 solution in DMSO (99.97%, H_2_O < 0.1%) for 15 minutes at room temperature. The cells were analyzed using flow cytometry (Beckman Coulter Instrument FC 500) with a 488 nm argon laser using the MXP software Beckman Coulter program and the results are presented as the percentage of cells with depolarized mitochondria. Statistical analyses were performed using the STATISTICA 10.0 software package (StatSoft, Inc. (2010) version 10.0. http://www.statsoft.com). Normality was checked using the Kolmogorov-Smirnov test. The data were tested for homogeneity of variance using Levene’s test of equality of error variances. The significance of the differences in the levels of the percentage of cells with depolarized mitochondria between the intestine and hepatopancreas was assessed using the Student’s t-test, *p<0*.*05*. The significance of differences between the experimental groups in the levels of the analyzed parameter in relations to the time of starvation or re-feeding was assessed using the Tukey’s test for unequal sample size at *p < 0*.*05*.

#### JC1 staining—Mitochondrial membrane potential for confocal microscopy

The prepared intestine and hepatopancreas that were isolated from the shrimp (Table 2) were incubated in the dark with 5 μL of 1.5 mM JC-1 solution in DMSO (99.97%, H_2_O < 0.1%) for 15 minutes at room temperature without fixation. Next, the material was washed several times with PBS. The nuclei were stained for 10 min in 1 mg ∙ ml^-1^ Hoechst 33342 diluted in PBS and washed several times in PBS. The material was visualized using an Olympus FluoView FV1000 confocal microscope.

#### Point of No-Return (PNR_50_), regeneration

PNR_50_ –point of no-return is defined as a point in time during starvation when the daily mortality that is achieved in the population is maximal. After this point, regeneration is not possible. In order to obtain the value of the point of no-return (PNR_50_), mortality data was fitted using the Boltzmann sigmoid function. The initial and final values of the sigmoid were fixed to 0 and 100% (this means that all of the shrimp are alive at the beginning of experiment and all will die at the end of experiment The data points that were gathered from 22 days were used to perform the fit. Regeneration time was estimated as the time from the start of re-feeding to the time when the increase in mortality stopped.

## Supporting information

S1 Video 3D representation of mitochondrial transmembrane potential in the hepatopancreas of *N*. *davidi* exposed to 7 days of starvation. Active mitochondria with a high membrane potential (red signals), inactive mitochondria with a low membrane potential (green signals), nuclei (blue signals). JC-1 staining. Hoechst 33342 staining. Confocal microscope.
(MP4)

S2 Video 3D representation of mitochondrial transmembrane potential in the intestine of *N*. *davidi* exposed to 7 days of starvation Active mitochondria with a high membrane potential (red signals), inactive mitochondria with a low membrane potential (green signals), nuclei (blue signals). JC-1 staining. Hoechst 33342 staining. Confocal microscope.
(MPG)

S3 Video 3D representation of mitochondrial transmembrane potential in the hepatopancreas of *N*. *davidi* exposed to 14 days of starvation. Active mitochondria with a high membrane potential (red signals), inactive mitochondria with a low membrane potential (green signals), nuclei (blue signals). JC-1 staining. Hoechst 33342 staining. Confocal microscope.
(MPG)

S4 Video 3D representation of mitochondrial transmembrane potential in the intestine of *N*. *davidi* exposed to 14 days of starvation. Active mitochondria with a high membrane potential (red signals), inactive mitochondria with a low membrane potential (green signals), nuclei (blue signals). JC-1 staining. Hoechst 33342 staining. Confocal microscope.
(MPG)

S5 Video 3D representation of mitochondrial transmembrane potential in the hepatopancreas of *N*. *davidi* exposed to 21 days of starvation. Active mitochondria with a high membrane potential (red signals), inactive mitochondria with a low membrane potential (green signals), nuclei (blue signals). JC-1 staining. Hoechst 33342 staining. Confocal microscope.
(MPG)

S6 Video 3D representation of mitochondrial transmembrane potential in the intestine of *N*. *davidi* exposed to 21 days of starvation. Active mitochondria with a high membrane potential (red signals), inactive mitochondria with a low membrane potential (green signals), nuclei (blue signals). JC-1 staining. Hoechst 33342 staining. Confocal microscope.
(MPG)

S7 Video 3D representation of mitochondrial transmembrane potential in the hepatopancreas of *N*. *davidi* 4 days re-fed after 14 days of starvation. Active mitochondria with a high membrane potential (red signals), inactive mitochondria with a low membrane potential (green signals), nuclei (blue signals). JC-1 staining. Hoechst 33342 staining. Confocal microscope.
(MPG)

S8 Video 3D representation of mitochondrial transmembrane potential in the intestine of *N*. *davidi* 4 days re-fed after 14 days of starvation. Active mitochondria with a high membrane potential (red signals), inactive mitochondria with a low membrane potential (green signals), nuclei (blue signals). JC-1 staining. Hoechst 33342 staining. Confocal microscope.
(MPG)

S9 Video 3D representation of mitochondrial transmembrane potential in the hepatopancreas of *N*. *davidi* 7 days re-fed after 14 days of starvation. Active mitochondria with a high membrane potential (red signals), inactive mitochondria with a low membrane potential (green signals), nuclei (blue signals). JC-1 staining. Hoechst 33342 staining. Confocal microscope.
(MPG)

S10 Video 3D representation of mitochondrial transmembrane potential in the intestine of *N*. *davidi* 7 days re-fed after 14 days of starvation. Active mitochondria with a high membrane potential (red signals), inactive mitochondria with a low membrane potential (green signals), nuclei (blue signals). JC-1 staining. Hoechst 33342 staining. Confocal microscope.
(MPG)

S11 Video 3D representation of mitochondrial transmembrane potential in the hepatopancreas of *N*. *davidi* 14 days re-fed after 14 days of starvation. Active mitochondria with a high membrane potential (red signals), inactive mitochondria with a low membrane potential (green signals), nuclei (blue signals). JC-1 staining. Hoechst 33342 staining. Confocal microscope.
(MPG)

S12 Video 3D representation of mitochondrial transmembrane potential in the intestine of *N*. *davidi* exposed 14 days re-fed after 14 days of starvation. Active mitochondria with a high membrane potential (red signals), inactive mitochondria with a low membrane potential (green signals), nuclei (blue signals). JC-1 staining. Hoechst 33342 staining. Confocal microscope.
(MPG)

S1 Abstract Congress of progress in cell biology: mitochondria and chloroplasts (Poland, 2015). (TIF)

S2 Abstract XXXII conference on embryology plants, animals, humans (Poland 2016). (TIF)
