# Supplementary figures and images for: The effect of starvation and re-feeding on mitochondrial potential in the midgut of Neocaridina davidi (Crustacea, Malacostraca)
Source: PLoS One. 2017 Mar 10;12(3):e0173563. doi: 10.1371/journal.pone.0173563 (PMC5345833; doi:10.1371/journal.pone.0173563)

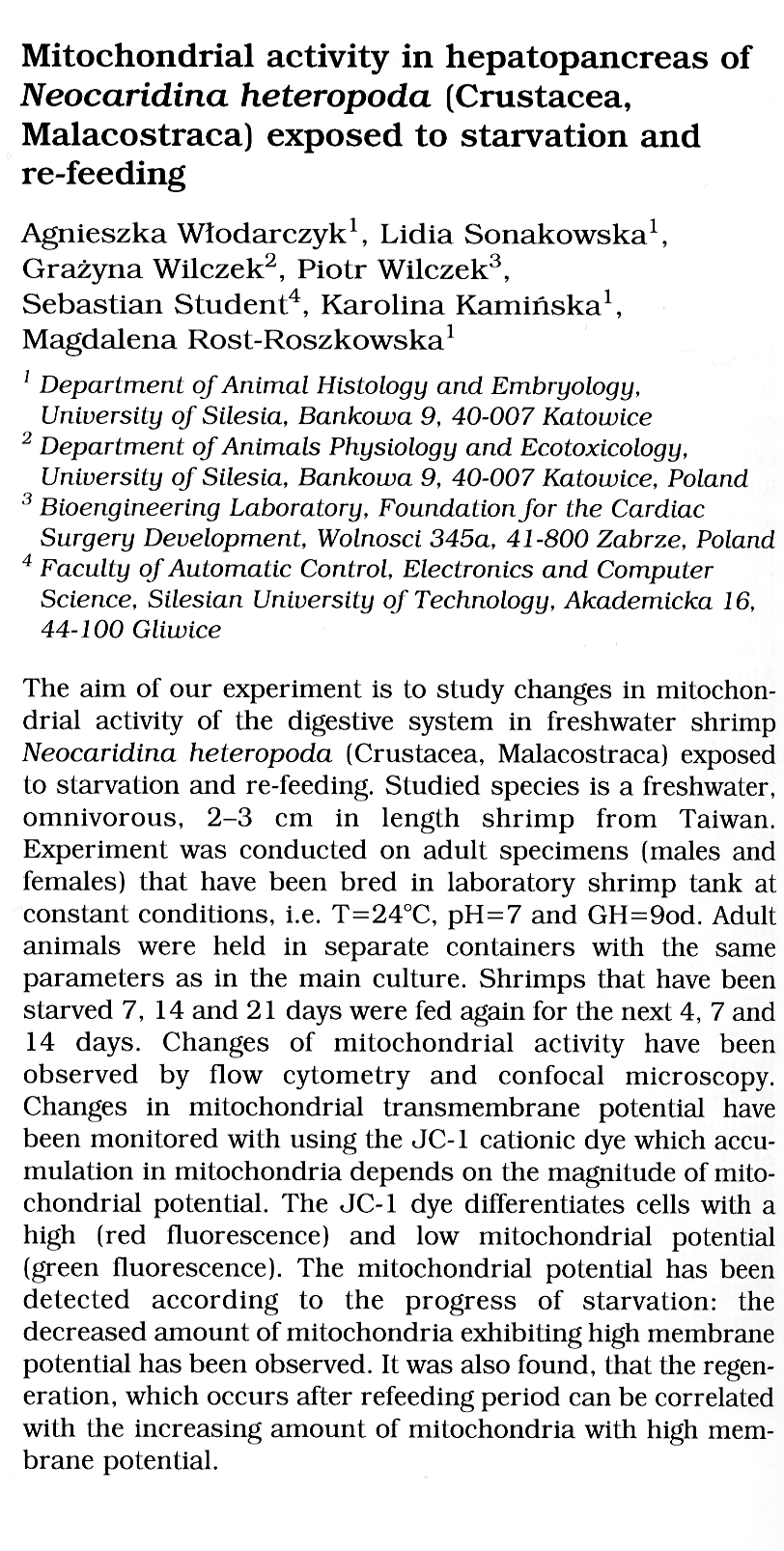

Supplement: S2 Abstract — (TIF) [file pone.0173563.s014.tif]
